# Identification of an Emergent Porcine Circovirus-2 in Vaccinated Pigs from a Brazilian Farm during a Postweaning Multisystemic Wasting Syndrome Outbreak

**DOI:** 10.1128/genomeA.00163-14

**Published:** 2014-03-20

**Authors:** Rafael L. Salgado, Pedro M. P. Vidigal, Luiz F. L. de Souza, Thiago S. Onofre, Natália F. Gonzaga, Monique R. Eller, Gustavo C. Bressan, Juliana L. R. Fietto, Márcia R. Almeida, Abelardo Silva Júnior

**Affiliations:** a Laboratory of Animal Molecular Infectology, Universidade Federal de Viçosa, Campus da UFV, Viçosa, Minas Gerais, Brazil; b Laboratory of Bioinformatics, Institute of Biotechnology Applied to Agriculture (BIOAGRO), Universidade Federal de Viçosa, Campus da UFV, Viçosa, Minas Gerais, Brazil; c Department of Food Technology, Universidade Federal de Viçosa, Campus da UFV, Viçosa, Minas Gerais, Brazil

## Abstract

Three porcine circovirus-2 strains were isolated from pigs on a Brazilian farm during an outbreak, indicating a vaccine failure. They present identical genomic sequences, with high identities to other isolates that were also related to vaccination failures, supporting the recent theory about an antigen drift being associated with vaccine failures throughout the world.

## GENOME ANNOUNCEMENT

Porcine circovirus-2 (PCV2) belongs to the family *Circoviridae*, genus *Circovirus*, and was initially identified as the causative agent of postweaning multisystemic wasting syndrome (PMWS) (1, 2). Since its identification, PMWS has become an endemic syndrome in most pig-producing countries, and PCV2 has reached a worldwide distribution (3, 4).

The porcine circovirus-2 genome is ambisense and composed of single-stranded circular DNA, with at least three open reading frames (ORFs). ORF1 encodes viral replication proteins (*rep* and *rep*′) (5), ORF2 encodes a capsid structural protein (*cap*), and ORF3 encodes an apoptosis-related protein (6). The PCV2 isolates currently are classified into three major genotypes: PCV2a, PCV2b, and PCV2c (7).

In Brazil, PMWS was first described in 2003 (8), where viral isolates of genotype PCV2b are prevalent (9), and retrospective studies have identified PCV2 in archived tissue samples dated from 1978 (10). Nevertheless, vaccination against PCV2 was only introduced in Brazil after 2007 as a measure to control PMWS.

Here, we present the complete genomic sequence of a new PCV2 strain (PCV2-UFV1) isolated from pigs on farms located in southeast Brazil during a PMWS outbreak in September 2013. Surprisingly, all animals were vaccinated against PCV2, indicating a vaccine failure, which had not yet been described for Brazilian farms.

Tissue samples from nine animals were collected, and PCV2 infection was confirmed by PCR using two pairs of primers, which amplify the full-length genome of PCV2 (1NF [5′-GGACCCCAACCCCATAAAA-3′] and 1NR [5′-CCCTCACCTATGACCCCTATGT-3′], and 2NF [5′-TGTTTTCGAACGCAGTGCC-3′] and 2NR [5′-CCGTTGTCCCTGAGATCTAGGA-3′]). The PCR products were purified and sequenced by Macrogen, Inc. (Seoul, South Korea). The complete genomic sequence is composed of 1,767 nucleotides (nt) and has a 48.4% G+C content, presenting the three major ORFs that are characteristic of the PCV2 genome. For comparison purposes, 1,126 genomic sequences of PCV2 isolates were downloaded from GenBank (http://www.ncbi.nlm.nih.gov/Genbank), including the reference isolates of genotypes PCV2a (accession no. AF055392), PCV2b (accession no. AF055394), and PCV2c (accession no. EU148503). The genomic sequences were aligned using MAFFT (11), and a pairwise distance matrix was calculated using MEGA (12).

The genotyping of PCV2-UFV1 was performed as described by Vidigal et al. (3), and this virus was classified as belonging to the PCV2b genotype. In pairwise genomic comparisons, its genome showed high identities (>99%) with 154 other viral isolates from China, Serbia, South Korea, and the United States. Among the isolates, the Chinese BDH strain (accession no. HM038017) is associated with antigenic changes in PCV2 (13), and the North American US22625-33 (accession no. JX535296) and US22664-35 (accession no. JX535297) strains are associated with vaccine failures (14). Further analysis of ORF2 revealed that PCV2-UFV1 shares a similar set of amino acid substitutions that are related to antigenic changes in the BDH, US22625-33, and US22664-35 strains, including an additional lysine residue at position 234 (13, 14).

We have reported a new Brazilian strain (PCV2-UFV1) isolated from vaccinated pigs during a PMWS outbreak, which is similar to isolates associated with outbreaks and vaccination failures in China and the United States. These data support the recent theory on the occurrence of an antigenic drift of PCV2 that might be the cause of vaccine failures throughout the world.

### Nucleotide sequence accession number.

The complete genome sequence of PCV2-UFV1 has been deposited in GenBank under the accession no. KJ187306.
